# Quantity and quality are increasing but there's room for improvement: A scoping review of physical activity intervention trials

**DOI:** 10.1016/j.bjpt.2024.101051

**Published:** 2024-03-23

**Authors:** Marina B Pinheiro, Ana Helena S Reis, Jennifer N Baldwin, Anne M Moseley, Vishwesh Bapat, Courtney S Chan, Wing S. Kwok, Catherine Sherrington

**Affiliations:** a Sydney Musculoskeletal Health, The University of Sydney, Sydney, Australia; b Institute for Musculoskeletal Health, Sydney Local Health District, Sydney, Australia; c School of Public Health, Faculty of Medicine and Health, The University of Sydney, Sydney, Australia; d Departamento de Fisioterapia, Faculdade Ciências Médicas de Minas Gerais, Minas Gerais, Brazil; e WHO Collaborating Centre for Physical Activity, Nutrition and Obesity, Charles Perkins Centre, The University of Sydney, Australia

**Keywords:** Evidence-based practice, Physical activity, Quality, Risk of bias, Trial

## Abstract

**Background:**

Observing trends in research publications helps to identify the quantity and quality of research produced, as well as reveal evidence gaps. No comprehensive review of the quality and quantity of physical activity intervention trials has been conducted.

**Objective:**

We aimed to investigate i) the volume and quality (and changes in these over time) of randomized controlled trials evaluating physical activity interventions, and ii) the association between journal ranking and trial quality.

**Methods:**

We searched the Physiotherapy Evidence Database (PEDro) for trials investigating physical activity interventions (no restrictions for population, comparison, or language). Descriptive statistics were used to describe the volume and quality of trials. The association between journal ranking (Journal Impact Factor) and trial quality (PEDro Scale) was examined using Spearman's rho correlation.

**Results:**

We identified 1779 trials, of which 40% (*n* = 710) were published between 2016 and 2020. The mean (SD) total PEDro score was 5.3 (1.5) points out of 10, increasing over time from 2.5 (0.7) points in 1975–1980 to 5.6 (1.4) points in 2016–2020. Quality criteria that were least reported included blinding of intervention deliverers (therapists) (*n* = 3, 0.2%), participants (*n* = 21, 1.2%), or assessors (*n* = 541, 31%); concealed allocation to groups (*n* = 526, 30%); and intention to treat analysis (*n* = 764, 43%). There was a small correlation between trial quality and Journal Impact Factor (0.21, *p* < 0.001).

**Conclusion:**

A large volume of trials has investigated physical activity interventions. The quality of these trial reports is suboptimal but improving over time. Journal ranking should not be used for selecting high quality trials.

## Highlights

• There are 1779 physical activity trials in the physiotherapy evidence database (PEDro).
• The mean (SD) PEDro score was 5.3 (1.5) points out of 10, reflecting ‘fair to poor’ quality.
• Journal impact factor is weakly correlated with trial quality (0.21, *p* < 0.001).
• We make five recommendations to improve future trial quality.

## Introduction

Physical inactivity is linked with at least 5 million deaths and more than $67 billion of economic burden globally each year.^1^^,^^2^ The World Health Organization (WHO) recommends that people of all ages and abilities engage in regular physical activity.^3^ However, despite massive growth in physical activity research in recent decades, population physical activity levels have remain largely unchanged.^4^ Understanding the current quality of evidence for physical activity interventions is critical to inform priority areas for future research and resource allocation.

Examining trends in research publications helps to identify the quantity and quality of research produced, as well as reveal evidence gaps.^5^ A bibliometric review of a random sample of 4069 physical activity studies found that descriptive studies remain the most common study type, with far fewer publications reporting on the efficacy of physical activity interventions or dissemination studies.^6^ However, the review described trends over three time periods only (2008–2009, 2013–2014 and 2017–2018).^6^ Bibliometric reviews have been undertaken for physical therapy trials in general^7^, ^8^, ^9^ as well as for cardiothoracic,^10^ neurological,^11^ and musculoskeletal interventions.^12^^,^^13^ However, to our knowledge, no comprehensive review of the quality and quantity of physical activity intervention studies has been conducted.

Heterogeneity in trial quality poses a barrier for clinicians to incorporate evidence into practice, with poor quality trials being more likely to provide biased estimates of effects than good quality trials.^14^^,^^15^ High quality research is generally more likely to be published in high ranking journals,^16^ and while journal ranking can be quantified using Journal Impact Factor, this metric has its limitations.^17^^,^^18^ Nevertheless, a strong relationship between journal ranking and trial quality could help clinicians and researchers to use this metric to identify high quality trials. Previous reviews of trials investigating psychiatric and hepatobiliary interventions found no association between journal ranking and trial quality.^19^^,^^20^ However, it remains unclear whether journal ranking - measured by Journal Impact Factor - is associated with individual study quality for trials investigating physical activity interventions.

In this review we focus on interventions targeting overall physical activity levels (rather than specific therapeutic exercise) considering the launch of the WHO Global Action Plan on Physical Activity in 2018.^21^ The primary aim of this review was to describe the volume, quality, and changes in volume and quality over time of randomized controlled trials evaluating the effects of physical activity interventions. The secondary aim was to investigate whether there was a relationship between trial quality and journal ranking as measured by Journal Impact Factor.

## Methods

### Data sources and search

As our aim was to explore the breadth of the body of literature and identify evidence gaps, a scoping review was chosen as the preferred method.^22^ We conducted a scoping review following the Joanna Briggs Institute scoping review methods guideline^23^ and the Preferred Reporting Items for Systematic reviews and Meta-Analyses extension for Scoping Reviews (PRISMA-ScR) Checklist.^24^ We registered our protocol a priori and published this on the Open Science Framework website.^25^

We searched the Physiotherapy Evidence Database (PEDro; pedro.org.au) from inception to December 2020 using the following text words as phrases: “physical activity”, “step count”, “sedentary behavior” (also behavior), and “sedentary time” in the “title and abstract” field. Only randomized controlled trials were included, and no date or language limits were applied. The search strategy is detailed in Supplementary material A.

We selected PEDro as the data source because it is the most comprehensive database indexing randomized controlled trials of any intervention that is or could be relevant for physical therapists worldwide, including physical activity interventions.^26^, ^27^, ^28^ The PEDro database is constructed using a robust multifaceted search strategy, including automated optimized searches performed in several databases (Medline, Embase, CINAHL, PsycINFO, AMED, Cochrane Database of Systematic Reviews, Cochrane CENTRAL Register of Controlled Trials), citation tracking of systematic reviews indexed in PEDro, and trials identified by users.^28^ In 2021, PEDro users were from 215 countries, and the database had one new search being performed every 7 s.^29^ All trial reports in PEDro are coded for: therapy (e.g., behavior modification; education); problem (e.g., frailty; pain; reduced exercise tolerance); body part (e.g., head or neck; chest; ankle or foot); and area of physical therapy practice (e.g., cardiothoracics; continence and women's health; musculoskeletal). Each trial can receive up to three codes for each category. The full list of codes can be found in Supplementary material B.

The methodological quality of all trials indexed in PEDro is assessed using the 11-item PEDro scale.^30^ Item 1 (inclusion criteria and source of participants) relates to external validity and is not used to calculate the final score. Items 2 to 9 assess internal validity (allocation, blinding, completeness of follow-up, intention-to-treat analysis) and items 10 to 11 assess statistical reporting (between-group comparisons, mean, and variability data). The total PEDro score is calculated by summing items 2 to 11 and ranges from 0 to 10 points. As two items are for blinding of therapists and participants - which is rarely possible for physical activity interventions - it is generally accepted that the maximum score for trials evaluating physical activity interventions is 8/10. The total PEDro score as well as individual item scores are available for every trial indexed in PEDro. Previous studies have shown a high inter-rater agreement for the total PEDro score (Interclass Correlation Coefficient = 0.91),^31^ adequate agreement for individual items (Kappa = 0.52 to 1.00),^31^ and acceptable convergent and construct validity for 8 of the 10 items.^32^

### Study selection

Two independent reviewers screened potentially relevant articles based on titles and abstracts. A third reviewer was consulted in case of disagreements. The number of included articles and reasons for exclusion were recorded.

We only included articles reporting the results of randomized controlled trials investigating the effects of physical activity interventions aimed at improving physical activity levels or participation in any population or comparison group. The WHO's broad definition of physical activity was used, where “physical activity is defined as any bodily movement produced by skeletal muscles that requires energy expenditure.”^33^ Trials investigating any form of whole-body physical activity intervention were included, provided that physical activity level (e.g., physical activity level or duration, number of steps, time spent in sedentary activities, and energy expenditure assessed by questionnaire or accelerometer) and/or participation were specified as outcomes. Trials investigating passive strategies to increase physical function (such as manual therapy and electrical therapy) and interventions targeted at one specific body part (such as isolated muscle activity) were excluded.

### Data extraction and methodological quality

The following information were extracted from PEDro for all included trials: area of physical therapy practice (Supplementary material B), year of publication, language of publication, journal name, and PEDro scores (total PEDro scores /10 as well as the achievement of individual items). To indicate trial quality, a cut-off point of 6 points or higher was used to reflect ‘good’ methodological quality, with scores below 6 points considered as ‘fair to poor.’^34^ We used Journal Impact Factor to quantify journal ranking. The 2020 Journal Impact Factors of eligible papers were downloaded from Web of Science (Journal Citation Reports).

### Analysis

We used descriptive statistics to quantify volume and quality of the included trials: the number of trials, year of publication, number of trials according to language and area of physical therapy practice, scoring of individual PEDro scale items, and total PEDro score. We explored the quality of trials according to areas of physical therapy practice and language of publication. We investigated changes over time by analyzing the volume and quality (total PEDro score and individual items) of physical activity trials according to year of publication from 1975 to 2020, divided into 5-year intervals, except for the first interval, which was 6 years (1975–1980, 1981–1985, until 2016–2020). We investigated the association between trial quality (total PEDro score) over time (year of publication) using simple regression analysis. The association between journal ranking (Journal Impact Factor) and trial quality (total PEDro score) was examined using Spearman's rho correlation due to non-normal data distribution. All data were analyzed using Microsoft Excel for Mac version 16 and STATA version 16. Statistical significance was set at *p* *<* *0.05.*

## Results

### Volume of physical activity trials

**Fig. 1:**
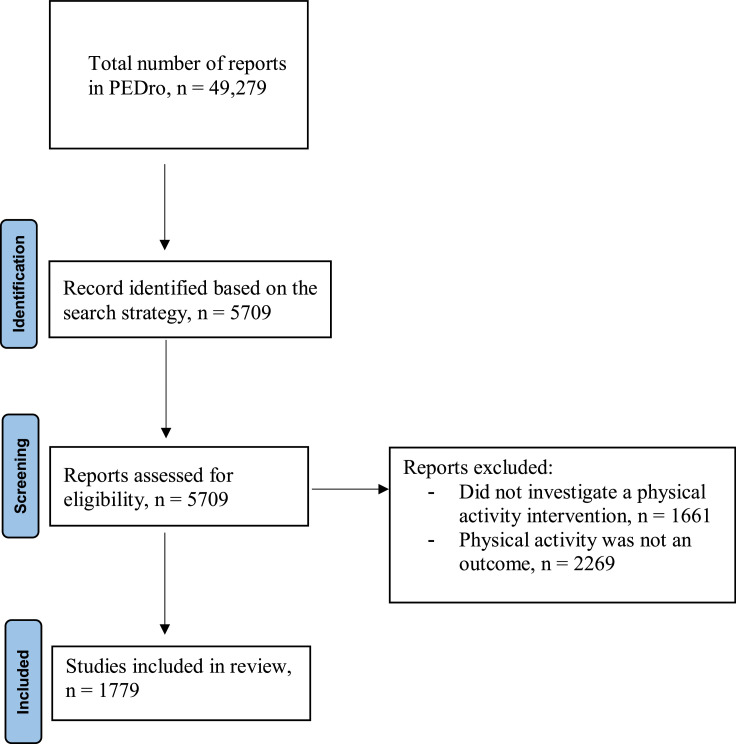
PRISMA flow diagram of study selection.

**Fig. 2:**
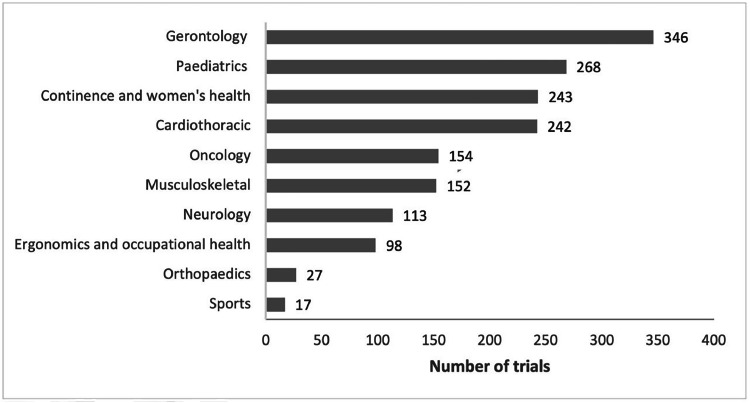
Number of articles reporting randomized controlled trials of physical activity interventions according to areas of practice of physical therapy. Note: as each trial can be classified for more than one area of practice, the total number of articles in this graph does not match the total number of articles included in this review.

The search identified 5709 articles and, after title and abstract screening, a total of 1779 articles were included (Fig. 1). Most articles were published in English (*n* = 1755, 99%). The remaining 24 articles were published in nine different languages (German, Japanese, Korean, Persian, Polish, Spanish, Chinese, Icelandic, Russian). The most common areas of physical therapy practice were gerontology (*n* = 346) and pediatrics (*n* = 268) (Fig. 2). There were 372 trials that were classified as “other” for area of physical therapy practice (e.g., general population, obesity, mental health, and diabetes).

**Fig. 3:**
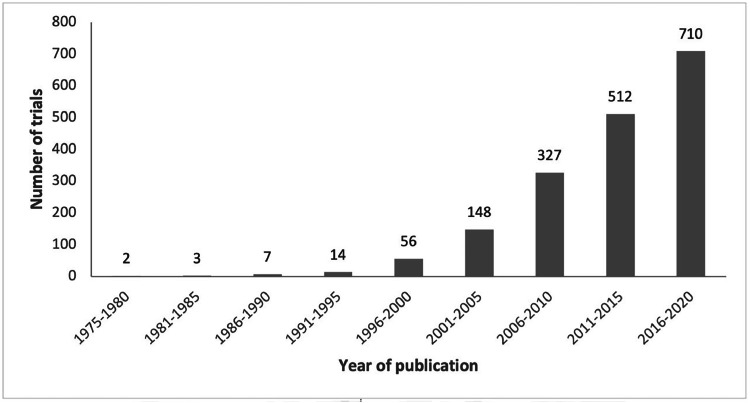
Number of articles reporting randomized controlled trials of physical activity interventions indexed in the Physiotherapy Evidence Database (PEDro) according to year of publication displayed in 5-year intervals (except for the first interval which is 6 years).

Since publication of the first article in 1975, the number of trials has increased, with 40% (*n* *=* 710) of included articles published in 2016–2020 (Fig. 3). There was a 22-fold increase in publications in the final 20-year period, from 82 trials in 2001 to 1779 trials in 2020. There was a statistically significant improvement in quality over time, regression coefficient = 0.06 (95% confidence interval 0.05, 0.07), *p* *<* 0.001*.*

### Quality of articles reporting the results of physical activity trials

**Fig. 4:**
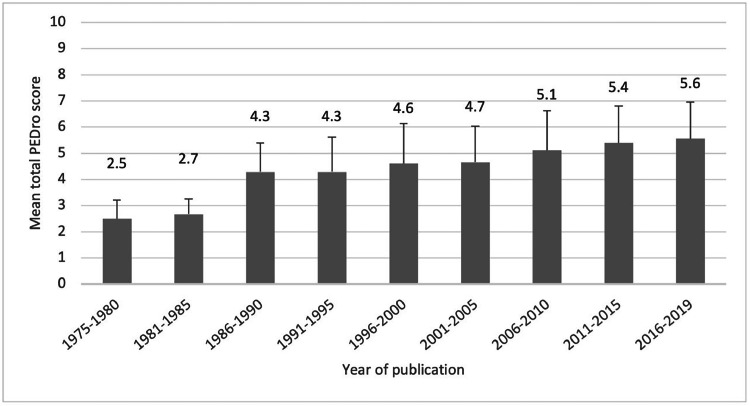
Total PEDro score (mean and SD) of articles reporting randomized controlled trials investigating physical activity interventions indexed in the Physiotherapy Evidence Database (PEDro) displayed in 5-year intervals (except for the first interval which is 6 years). The mean PEDro score for each period is indicated at the top of each bar. The error bar indicates the standard deviation for each period. Note: this graph is based on ratings for 1773 trials with complete data. The remaining 6 trials currently do not have a rating in PEDro and they were published in Persian (*n* = 4), Icelandic (*n* = 1), and German (*n* = 1).

The mean (SD) total PEDro score of the included articles was 5.3 (1.5) points out of 10, which reflects fair methodological quality. The mean PEDro score has increased over time, from 2.5 (0.7) in 1975–1980 to 5.6 (1.4) in 2016–2020 (Fig. 4). Despite the continuous improvement in quality observed over the years, about half of the articles (350/708, 49%) published between 2016 and 2020 would be classified as poor or fair quality (<6 points).

**Fig. 5:**
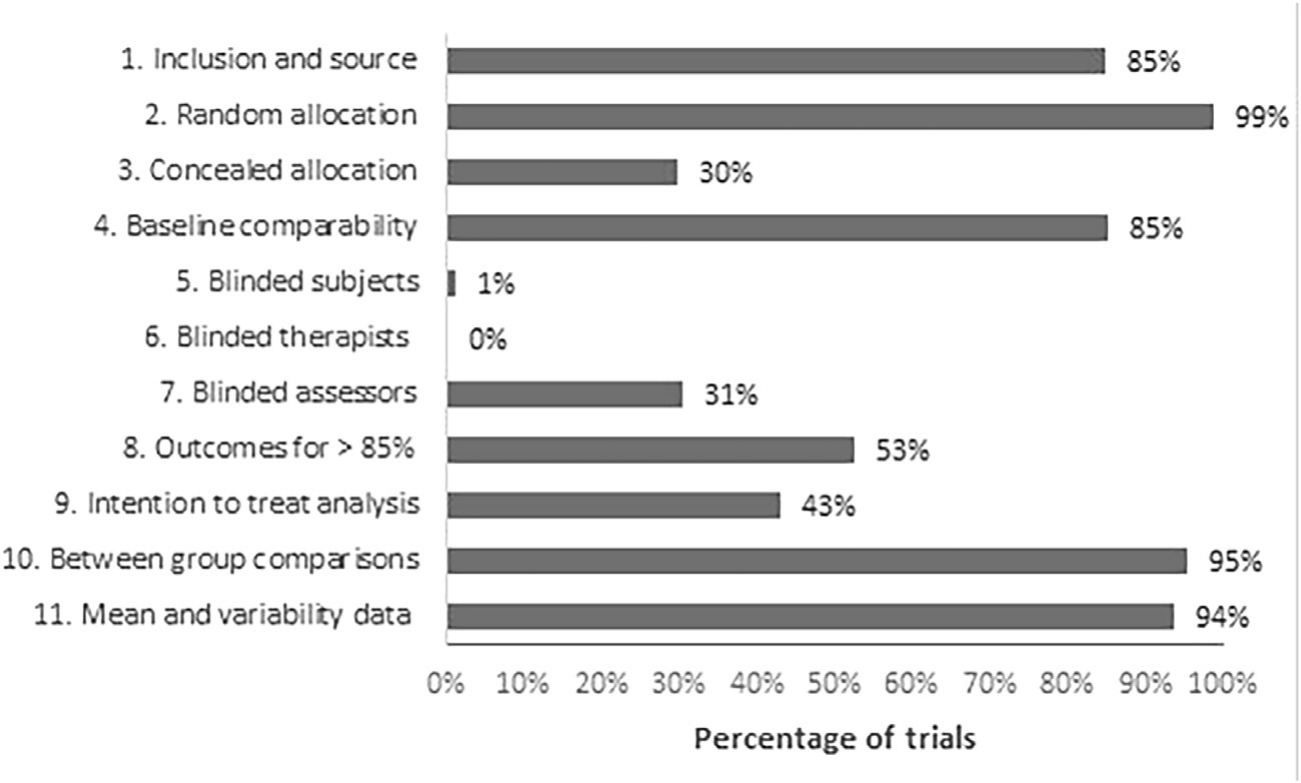
Percentage of articles reporting randomized controlled trials indexed in the Physiotherapy Evidence Database (PEDro) that satisfy each item of the PEDro scale for all trials published from 1975 to 2020.

The percentage of articles fulfilling each item of the PEDro scale is displayed in Fig. 5. Most articles used random allocation (*n* = 1751, 99%) and had similar groups at baseline regarding the most important prognostic indicators (*n* = 1510, 85%). Articles infrequently reported blinding of therapists (*n* = 3, 0.2%), participants (*n* = 21, 1.2%), or assessors (*n* = 541, 31%); concealed allocation (*n* = 526, 30%); and intention to treat analysis (*n* = 764, 43%). Approximately half (53%) of articles had outcomes for more than 85% of participants. Overall, the statistical reporting of the articles was good, with most articles reporting between-group comparisons (95%) and providing point estimates and measures of variability of data (94%). When comparing articles published in the last 5-year interval (2016–2020) with those published before 2016, the percentage of articles fulfilling each item of the PEDro scale had a small increase, particularly for concealed allocation, baseline comparability, and blinded assessors. In terms of external validity, most articles (85%) reported source and inclusion criteria.

When stratified by area of physical therapy practice, the mean (SD) PEDro score varied from 4.9 (1.5) points for ergonomics and occupational health to 6.2 (1.3) points for orthopedics. The mean PEDro score was below the cut-off threshold of 6 to identify “good quality trials” for most areas, with exception for orthopedics and neurology (Supplementary material C).

### Association between journal impact factor and trial quality

There were 122 articles that were published in journals without a Journal Impact Factor, and these were excluded from this analysis. Based on 1655 articles, there was a small correlation between journal ranking (Journal Impact Factor) and trial quality (total PEDro score), with a Spearman's rho correlation of 0.21 (*p* < 0.001).

## DISCUSSION

We identified a rapidly growing volume of randomized clinical trials investigating physical activity interventions. Trial quality has been slowly improving over the years but remains suboptimal, with the mean PEDro total score corresponding to ‘fair’ methodological quality. Commonly reported limitations of these trials included lack of assessor blinding (we acknowledge that blinding of therapists and participants is rarely possible), allocation concealment, intention-to-treat analysis, and a high loss to follow-up. These findings suggest that there is still great potential to improve the quality of physical activity trials. There was a small association between journal ranking and trial quality, suggesting that Journal Impact Factor alone should not be used for identifying high quality physical activity trials.

### Comparison with previous studies

Previous bibliometric analyses show that most physical activity studies are observational, with intervention studies accounting for one-quarter of publications (a proportion that has not increased over time).^6^^,^^35^ In contrast, our review found a large and growing body of evidence of physical activity intervention studies. However, the types of intervention trials conducted remain unclear, as this was beyond the scope of our review. To optimize impact on health outcomes and facilitate translation of results into practice, intervention research must move beyond efficacy studies (evaluating the impact of an intervention under optimal conditions) to replication (assessing the practicality of implementing efficacious interventions under real world conditions) and dissemination studies (investigating widespread use of an evidence-based intervention across a whole population).^36^^,^^37^ Future studies could investigate the types of intervention studies used in physical activity trials as this would further guide the research agenda and help identify priorities.

### Comparison with other fields

The mean total PEDro score of 5.3 points for physical activity trials in our review was very similar to the mean score for all trials indexed in PEDro (5.2 points, all trials up to February 2022),^29^ and for trials of interventions for low back pain (5.4 points, all trials up to July 2019)^13^ and musculoskeletal conditions (5.3 points, random sample selected in May 2017).^12^ We found that blinding (participants, therapists, and assessors), concealed allocation, intention-to-treat analysis, and completeness of follow-up had the lowest percentage of articles fulfilling these PEDro criteria. These findings are largely in keeping with previous studies investigating the quality of all trials included in PEDro,^7^^,^^28^ as well as neurology,^11^ sports,^38^ low back pain,^13^ and cardiothoracic.^10^ The percentage of trials of physical activity that performed intention-to-treat analysis (43%) was higher than previous investigations (range from 14% for sports^7^ to 35% for low back pain^13^).

In our review, blinding of participants and therapists was very rare. This was expected, as it is challenging to blind participants and therapists for physical activity interventions. However, it is possible to blind assessors in trials where physical activity is not self-reported. It is also possible to conceal allocation and to perform intention-to-treat analyses in all trials.

### Recommendations for future trials of physical activity interventions

A better understanding of the volume and main methodological flaws and limitations of physical activity trials indexed in PEDro allowed us to identify aspects of methodological quality that need improvement and make specific recommendations for future physical activity research. These recommendations are discussed below and summarized in Table 1.

**Table 1:** Recommendations for the conduct and reporting of future trials of physical activity interventions.

1) **Conceal allocation of participants to groups** - Use an on-line system (e.g., Research Electronic Data Capture [REDCap]) that enables a record to be audited. - Have different people in different places generate the randomization sequence and recruit participants (e.g., telephone, email, or web-based randomization service). - Have the holder of the randomization sequence insert the allocation into sealed, opaque, and consecutively numbered envelopes to be opened after each participant has been enrolled in the trial.^39^ 2) **Analyze outcome data using an intention-to-treat approach** - Collect follow-up outcome measures for participants who do not receive their allocated treatment. - Describe in trial reports whether intention-to-treat analysis was used and how deviations from allocation were handled. - Make trial conclusions based on the results of the intention-to-treat analysis. 3) **Blind outcome assessment with objective tools** - Use objective measures (e.g., pedometers and activity monitors) and collect data using researchers who are blinded to group allocation. 4) **Maximize follow-up using trial methods and procedures** - Be clear about the demands involved during recruitment. - Collect multiple forms of contact information (email address, mobile number, address) from participants and family members. - Simplify outcome assessments in terms of number of follow-ups, difficulty, and length. Pilot test outcome assessment procedures. - Provide alternative methods to complete follow-up assessments (e.g., online, in-person). - Contact participants who have not completed their assessments. - Provide pre-paid and addressed envelopes for participants to return equipment (e.g., activity monitors) or paper-based questionnaires. - Provide incentives to complete assessments (e.g., small gifts), subject to ethical approval. 5) **Improve clarity and completeness of reporting** - Report source of participants as well as eligibility criteria. - Report simple demographics, prognostic variables, and outcomes at baseline for each group. - Use an estimation approach (i.e., the between-group difference and its 95% confidence interval) to report between-group comparisons.^40^

### Strengths and limitations

A strength of this review is the comprehensive investigation of all trials of physical activity interventions indexed in PEDro (*n* = 1779) without any limits to date or language of publication. However, while PEDro is the most comprehensive bibliographic database of trials of physical therapy interventions, indexing up to 99% of relevant studies,^41^^,^^42^ some trials may have been missed.

Due to the large number of articles included in this review, screening for inclusion was performed on the titles and abstracts. We did not collect data regarding type of physical activity intervention performed as this was beyond the scope of the review. Future studies could consider extracting additional information (e.g., sample size, type of intervention trial) as this could enhance our understanding of physical activity trials. We had initially planned to explore the volume and quality of physical activity trials based on language of publication, but because nearly all trials were published in English this analysis was not possible. Most trials indexed in PEDro are published in English.^28^ Future studies could consider searching databases in different languages, such as Wanfang Data for trials in Chinese and LILACS for trials in Portuguese and Spanish as this might increase the number of studies found. It is possible that our search strategy did not capture all physical activity trials, and future reviews could include a broader range of terms relevant to physical activity (e.g., exercise, physical fitness). Another potential limitation of this review is that the quality of trials was judged based on the reporting of the trial, rather than the trial conduct itself. It is possible that some trials put in place methods to minimize risk of bias but failed to report them and therefore this could not be accounted in the ratings of trial quality.

### Clinical implications

There are several implications for researchers and clinicians who need to incorporate physical activity evidence into practice. For researchers, the recommendations discussed in Table 1 can be implemented to minimize bias and improve trial reporting. Recommendations 1 to 4 cover methodological flaws that can be substantially improved by careful planning of trial methods and procedures (lack of concealed allocation, intention-to-treat analysis, blinding of assessor, and low follow-up rate). We acknowledge that greater resources in terms of time, personnel, and funding may also be needed to achieve blinding of assessors and maximize follow-up rates. The use of reporting guidelines, such as the CONSORT statement,^43^ would greatly help improving reporting the items eligibility criteria, source of participants, similarity of groups at baseline, and statistical reporting as well as allocation concealment and intention-to-treat analysis.

Based on the findings of this review, journal ranking alone should not be used for identifying articles reporting the results of physical activity trials. Instead, we recommend that clinicians and researchers use their searching and appraisal skills to identify and evaluate trials of physical activity interventions. Similar findings were observed in previous investigations within other clinical fields.^19^^,^^20^

## Conclusion

The volume of trials investigating physical activity interventions is substantial and growing. The reported quality of these trials is suboptimal but slowly improving over time. There is great potential for improvement in the quality of conduct and reporting of physical activity trials as most of the issues we identified can be easily remedied. The items ‘eligibility criteria’, ‘source of participants’, ‘similarity of groups at baseline’, and ‘statistical reporting’ would be improved merely by better reporting. Journal ranking should not be used alone for identifying high quality physical activity trials.

## Registration

This scoping review was registered on the Open Science Framework website, available at https://osf.io/73 × 5j/.

## Declaration of competing interest

Cathie Sherrington is a member of the PEDro Steering Committee. Anne Moseley was a member of the PEDro Steering Committee until December 2021. Marina B Pinheiro is a member of the PEDro training and development sub-committee. The remaining authors confirm they have no conflicts of interest.

## Appendix Supplementary materials

Image, application 1
