# Supplementary material for: Quantity and quality are increasing but there's room for improvement: A scoping review of physical activity intervention trials
Source: Braz J Phys Ther. 2024 Mar 23;28(2):101051. doi: 10.1016/j.bjpt.2024.101051 (PMC10999812; doi:10.1016/j.bjpt.2024.101051)
Supplement: Supplementary file 1 [file mmc1.pdf]

Supplementary material A. Search strategy – Physiotherapy Evidence Database (PEDro)

1. (physical* activity*) OR (sedentary* behaviour*) OR (sedentary* behavior*) OR (sedentary* time*) OR (step* count*) <title and abstract> field
2. …2020 <Year of Publication>
3. clinical trial <Method>
4. 1 AND 2 AND 3

Supplementary material B. Classification codes for randomised controlled trials indexed in the Physiotherapy Evidence Database (PEDro)

Source: PEDro website (https://pedro.org.au/english/learn/indexing-criteria-and-codes/)

Intervention

These categories cover broad aspects of physiotherapy intervention. If the intervention used in a particular paper does not fit under any of these, the code "no appropriate value in this field" should be used.

acupuncture
behaviour modification
education
electrotherapy, heat, cold
fitness training
health promotion
hydrotherapy, balneotherapy
neurodevelopmental therapy, neurofacilitation
orthoses, taping, splinting
respiratory therapy
skill training
strength training
stretching, mobilisation, manipulation, massage
no appropriate value in this field

Problem

This problem list has been developed to cover broad categories of problems treated by physiotherapists. Note that the problem has been described at the level of physiotherapy intervention. If the problem being treated in a particular paper does not fit under any of these, the code "no appropriate value in this field" should be used.

difficulty with sputum clearance
frailty
impaired ventilation
incontinence
motor incoordination
muscle shortening, reduced joint compliance
muscle weakness
oedema
pain
reduced exercise tolerance
reduced work tolerance

skin lesion, wound, burn
no appropriate value in this field

Body part
These categories refer to the body part being treated. If there is no one body part being treated, or if the intervention is targeted at whole body systems rather than a body part (eg, training walking following a stroke), the code "no appropriate value in this field" should be used.

head or neck
upper arm, shoulder, or shoulder girdle
forearm or elbow
hand or wrist
chest (for cardiothoracic trials)
thoracic spine
lumbar spine, SIJ, or pelvis
perineum or genito-urinary system
thigh or hip
lower leg or knee
foot or ankle
no appropriate value in this field

Subdiscipline
These categories refer to various aspects of physiotherapy. If the area for a particular paper does not fit under any of these, the code "no appropriate value in this field" should be used.

cardiothoracics
includes, but is not restricted to, papers evaluating acute and rehabilitation cardiothoracic interventions or fitness training on those with conditions affecting the cardiothoracic system. This subdiscipline does not include studies of general fitness training among patient populations.

continence and women's health
includes, but is not restricted to male and female incontinence and pre- and post-natal interventions for the mother

ergonomics and occupational health
includes, but is not restricted to, interventions based at workplaces or on workers for work-related conditions

gerontology
includes papers where the average age of the study sample is over 60, and papers on conditions which commonly affect older people (eg, arthritis)

musculoskeletal
includes, but is not restricted to, low back pain, rheumatoid disease, entrapment syndromes, neuralgia

neurology
includes, but is not restricted to, lesions of the central and peripheral nervous systems excluding those whose primary presentation is pain or paraesthesia such as carpal tunnel syndrome, neuralgia, or sciatica

oncology
includes papers evaluating interventions for health problems due to tumours or cancers

orthopaedics
includes only fractures and intervention before or after orthopaedic surgery (eg, knee replacements, ligament repairs)

paediatrics
includes papers where the average age of the study sample is under 16, and papers on conditions which commonly affect children (eg, cystic fibrosis)

sports
includes papers which specifically mention sports injuries as well as conditions which commonly affect sports people (eg, ligament repairs)

Supplementary material C. Total Physiotherapy Evidence Database (PEDro) scale score (mean and standard deviation) of articles reporting randomized controlled trials investigating physical activity interventions indexed in PEDro according to area of physical therapy.

The mean PEDro score for each physical therapy area is indicated at the top of each bar and the error bar indicates the standard deviation.

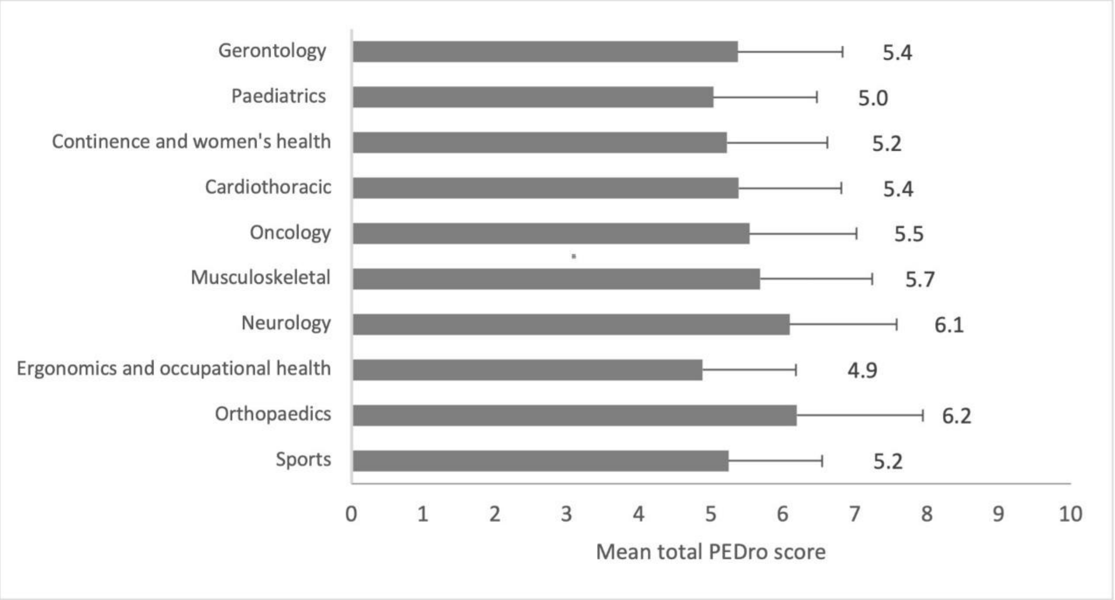

Note: this graph is based on ratings for 1773 trials with complete data.
